# Access to health services for international migrants during the COVID-19 pandemic: a qualitative study

**DOI:** 10.1590/1980-220X-REEUSP-2022-0443en

**Published:** 2023-08-14

**Authors:** Alice Blukacz, Báltica Cabieses, Alexandra Obach, Alejandra Carreño, Carolina Stefoni, Claudia Pérez

**Affiliations:** 1 Universidad del Desarrollo, Facultad de Medicina Clínica Alemana, Instituto de Ciencias e Innovación en Medicina, Santiago, RM, Chile.; 2 Universidad de Tarapacá, Facultad de Ciencias Sociales, Iquique, Tarapacá, Chile.; 3 Universidad del Desarrollo, Facultad de Medicina Clínica Alemana, Instituto de Ciencias e Innovación en Medicina y Carrera de Enfermería, RM, Chile.

**Keywords:** Human Migration, Health Services Accessibility, COVID-19, Chile, Migração Humana, Acesso aos Serviços de Saúde, COVID-19, Chile, Migración Humana, Accesibilidad a los Servicios de Salud, COVID-19, Chile

## Abstract

**Objective:**

To explore the experience and perception of international migrants in Chile regarding access to health services during the pandemic.

**Method:**

Collective case study following the qualitative paradigm. Forty semi-structured interviews were carried out with 30 migrants from different countries in Latin America and the Caribbean and 10 key actors from the health or social sector in November and December 2020. The interviews were analyzed thematically.

**Results:**

Perceived facilitators for general access to health services are related to formal work, support networks, and good treatment, while barriers are linked to immigration status, information gaps, discrimination, lack of cross-cultural skills, and personal limits of the system. In the context of access to COVID-19 diagnosis and treatment, the main barriers identified are: cultural approach to the disease, communication gaps, experiences of discrimination, costs, and lack of support networks.

**Conclusion:**

Access to health services is related to social vulnerability and violation of international migrants rights.

## RESUMEN

**Objetivo:**

Explorar la experiencia y percepción de las personas migrantes internacionales en Chile en torno al acceso a servicios de salud durante la pandemia.

**Método:**

Estudio de caso colectivo bajo el paradigma cualitativo. Se llevaron a cabo 40 entrevistas semi-estructuradas con 30 personas migrantes provenientes de diferentes países de América Latina y el Caribe y 10 actores clave del sector salud o social en noviembre y diciembre 2020. Se analizaron las entrevistas temáticamente.

**Resultados:**

Los facilitadores percibidos para el acceso general a servicios de salud se relacionan con el trabajo formal, las redes de apoyo y el buen trato, mientras que las barreras se vinculan con situación migratoria, brechas de información, discriminación, falta de competencias interculturales y límites propios del sistema. En contexto de acceso a diagnóstico y tratamiento de COVID-19, se identifican principalmente barreras: abordaje cultural de la enfermedad, brechas comunicacionales, experiencias de discriminación, costos y faltas de redes de apoyo.

**Conclusión:**

El acceso a servicios de salud se vincula con vulnerabilidad social y vulneración de derechos de las personas migrantes internacionales.

## RESUMO

**Objetivo:**

Explorar a experiência e a percepção dos migrantes internacionais no Chile sobre o acesso aos serviços de saúde durante a pandemia.

**Método:**

Estudo de caso coletivo sob o paradigma qualitativo. Foram realizadas 40 entrevistas semiestruturadas com 30 migrantes de diferentes países da América Latina e Caribe e 10 atores-chave do setor de saúde ou social em novembro e dezembro de 2020. As entrevistas foram analisadas tematicamente.

**Resultados:**

Os facilitadores percebidos para o acesso geral aos serviços de saúde estão relacionados ao trabalho formal, redes de apoio e bom tratamento, enquanto as barreiras estão ligadas ao status de imigração, lacunas de informação, discriminação, falta de habilidades interculturais e limites próprios do sistema. No contexto do acesso ao diagnóstico e tratamento da COVID-19, identificam-se principalmente barreiras: abordagem cultural da doença, lacunas de comunicação, vivências de discriminação, custos e falta de redes de apoio.

**Conclusão:**

O acesso aos serviços de saúde está vinculado à vulnerabilidade social e à violação dos direitos dos migrantes internacionais.

## INTRODUCTION

Access to health services by international migrants in transit in host countries is determined by multiple factors at the legal level, and at public policy, health system capacity, physical accessibility levels, and by individual factors, which are often explicitly or latently connected with experiences of social vulnerability^(1)^. International migrants are those outside their usual place of residence across an international border, excluding movements for leisure, vacation, visits to friends and family, business, medical treatment, or religious pilgrimage^(2)^. In 2020, it was estimated that there were some 281 million migrants worldwide, representing around 3.5% of the total population, 14.8 million of whom were in Latin America and the Caribbean^(3)^.

Migration is recognized as a social determinant of physical and mental health, since the migration process impacts it, from the pre-migration phase to the settlement phase in a host country, after stages of transit and initial arrival^(4)^. This, likewise, is connected to social vulnerability, which is experienced by a large proportion of people who move within the region, following South-South migration patterns with varying degrees of voluntary action in situations of armed conflict, and political, socioeconomic, and environmental crises. The COVID-19 pandemic has exacerbated the socioeconomic precariousness faced by migrants, as well as the risk of getting sick and the consequences it entails for people who often work in the informal sector and have limited support networks, making the need to ensure an inclusive and adequate response by the health system more urgent^(5,6)^. In Chile, migrants have reported different concerns about their health since the beginning of the pandemic that reveal interrelated layers of social vulnerability. On the one hand, they highlighted the need to rely on prevention measures appropriate to the reality they are facing, and on the other, the certainty of being able to be cared for in the health system regardless of their immigration status^(7)^. This second aspect turned out to be a very important element of differentiation with respect to the local population, since many aids provided by the State of Chile required having the national identification document.

International migrants in Chile currently represent 8% of the total population of the territory, about 1.5 million people^(8)^. The sharp increase in the Venezuelan population meant that it became the most represented nationality among international migrants in the country, replacing the Peruvian population. On the other hand, a diversification of nationalities and people from countries that are more geographically distant is observed^(9)^. A concerning aspect, however, is the increase in people entering through unauthorized crossings since the closure of the borders as a result of the COVID-19 pandemic. In 2021, the number of people entering the country through unauthorized passage registered in the north of the country reached 18,000^(10)^.

Regarding access to health, efforts have been made to break down barriers, among which Decree No. 67 of 2016, which allows free access to the public health system for those who have an irregular migratory status and do not receive an income^(11)^. However, 11% of the migrant population reports not being enrolled in either the public or the private health system^(12)^. Moreover, recent studies point to persistent barriers, where what is guaranteed by the Decree is not fulfilled in practice, or where barriers related to xenophobia, language, or lack of cultural relevance continue to hinder equal access to health care^(13)^. Ensuring effective access to relevant and culturally appropriate health care contributes to the coverage of the human right to health and represents an indispensable foundation for sustainable development^(14)^.

Therefore, the objective of this study is to explore the experience and perception of international migrants in Chile regarding access to health services during the pandemic. This allows identifying the main persistent or new facilitators and barriers, and thus develop a better response to health emergencies.

## METHODS

### Design of Study

Collective case study following the qualitative paradigm. This design was initially selected since it allows for in-depth investigation of the experience of the participants, taking into account their respective contexts and the processes experienced^(15)^. The research question guiding this analysis is the following: What are the facilitators and barriers to access to health services for international migrants during the COVID-19 pandemic in Chile, perceived by this population and by key actors in the health and social sector?

### Population and Local

Forty people participated, including 30 international migrants and 10 key actors from the health system or pro- migrant organizations. In terms of geographic coverage, the study was carried out in the Santiago Metropolitan Region and the Antofagasta and Arica and Parinacota regions in the north of the country. These three regions concentrate 75% of the migrant population nationwide.

### Inclusion and Exclusion Criteria

The inclusion criteria for the migrant population were the following: having been born outside Chile, being over 18 years of age, speaking Spanish, having lived in Chile during the pandemic, and living in one of the regions of interest. For the key actors, those of legal age, Spanish-speaking, and with experience in work with international migrants during the pandemic were considered. No specific exclusion criteria were applied.

### Sample and Recruitment

The participant selection process was carried out seeking diversity of experiences and discourses, thus including migrants of various nationalities, migratory status, socioeconomic status, and public/private health coverage. Participants recruitment was carried out in November and December 2020, simultaneously with the interview process. Both processes were carried out remotely, considering that in that period the vaccination campaign against COVID-19 had not yet started and mobility restrictions and social distancing recommendations were in force. Migrant participants were recruited from the extended networks of the research team and with the support of the Servicio Jesuita a Migrantes. The participants were contacted by phone and none refused to participate; however, three people did not connect to the video call at the agreed time and did not reschedule, giving lack of available time as the reason. The key actors were contacted by email and all agreed to participate.

### Data Collection Procedure

Individual semi-structured interviews lasting an average of 45 minutes were conducted through the Zoom platform or WhatsApp video call, from a place of convenience for the participant (home or workplace, in a secluded place to protect privacy). Each participant was interviewed only once.

Differentiated interview guides were used according to the characteristics of the person interviewed (migrant person or key actor). The interviews were recorded for later transcription, with the prior agreement of the participant. No field notes were taken and the interviews were not shared with the participants due to the time limits of the fieldwork. Following the preliminary analysis of the information, the research team agreed to have reached saturation after the 40 interviews, reaching sufficient information for the dimensions of barriers and facilitators of access to health care.

### Data Analysis

All interviews were transcribed *verbatim* and analyzed by AB, with subsequent validation by the research team. A thematic content analysis was carried out to identify patterns from the data, which were organized into codes and their respective subcodes.

### Ethical Aspects

The study was conducted according to the Declaration of Helsinki guidelines and was approved by the Ethical-Scientific Committee of the Universidad del Desarrollo (number 2020–117) before the beginning of the investigation. Participation in the project was completely voluntary and after receiving the relevant written information, all participants filled out an informed consent form through Google Forms, thus ensuring written digital consent. Furthermore, the participants were informed of their right to leave the study at any time or to refuse to answer any question during the interview, without consequences All the data were anonymized before their analysis and what is presented in this article does not allow the recognition of any participant personally. The coding that allows differentiating the citations of the migrant participants was established as follows: Sex Nationality Region Number (example: Peruvian woman RM 1). In the case of the key actors interviewed, they are only differentiated by a number (example: Key Actor 1).

### Data Availability

The data used in this study is available through the following link: http://doi.org/10.6084/m9.figshare.21493548

## RESULTS

### Sample Description

Sample composition of 30 migrant participants is described below (Table 1):

**Table 1.** Description of the sample of migrant participants – Santiago, Chile, 2020.

Variable	Frequency	Percentage
Age 1. 25-29 years 2. 30-35 years 3. 36-40 years 4. 41-45 years 5. over 45 years	12 9 4 3 2	40% 30% 13% 10% 7%
Sex 1. F 2. M	16 14	53% 47%
Region of residence 1. Metropolitan Region of Santiago 2. Arica and Parinacota 3. Antofagasta	22 6 2	73% 20% 7%
Nationality 1. Venezuela 2. Peru 3. Colombia 4. Haiti 5. Bolivia 6. Ecuador 7. Argentina 8. Brazil 9. Cuba 10. Uruguay	7 5 5 3 2 2 2 2 1 1	23% 17% 17% 10% 7% 7% 7% 7% 3% 3%
Migratory status 1. Visa pending 2. Current temporary visa 3. Permanent visa 4. Irregular status	11 5 9 4	37% 17% 30% 13%
Residence time in Chile 1. 6 months-1 year 2. 1-5 years 3. 6-10 years 4. Over 10 years	4 18 2 6	13% 60% 7% 20%
Healthcare coverage 1. FONASA (public) 2. ISAPRE (private) 3. does not have/does not know 4. Other	19 5 5 1	63% 17% 17% 3%

On the contrary, among the 10 key actors interviewed, 60% (6) identified themselves as men and the remaining as women. Half worked in the social sector and the other half in the health sector. Also, two were migrants, from Venezuela and Haiti.

#### Emergent Categories, Codes, Subcodes

**Figure 1.**
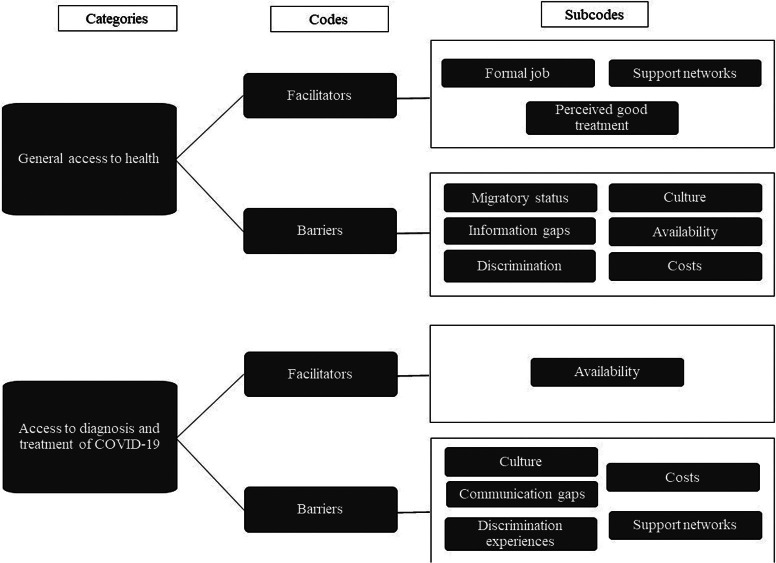
Emerging codes and subcodes.

After the thematic analysis, data and their respective emerging codes and subcodes were organized into categories, as described in Figure 1.

#### Context: Facilitators and General Barriers to Access to Health

First, it is important to describe the facilitators and barriers perceived by the participants when asked about their general experience with the Chilean health system.

Regarding the facilitators, the first thing that stands out is the formal work as a trigger for enrollment in the health system. Although people without formal resources, either migrants or Chileans, have the right to enroll in the public health system, not all of them do. However, several participants report having been enrolled by their employer. Additionally, the fact of having family members already registered led, in certain cases, to the registration and the decision to choose between the public or private system. *The truth is that I don’t remember exactly what section I am in, but I joined FONASA due to my first job, I worked in a school, as an assistant.* (Peruvian woman RM 1). Finally, several participants stressed the good treatment received in both public and private health centers. *In some centers the issue of health is different. I lived in Padre Orellana, by Vicuña Mackenna and the attention was expeditious, quiet, a small office, friendly doctors.* (Peruvian woman RM 2).

Nevertheless, the biggest barrier to accessing health care in the public system was that of immigration status, either because when going to a public health center they were denied care or because they avoided going out of fear of being turned in for their irregular status or for having the perception of not having the right to health care, something that was especially reported by the key actors interviewed and is linked to the lack of information about the rights of migrants regarding health. *My case is that when my son arrived here, he jumped from the second rung of the stairs, so I had to go and they didn’t want to see me, at that time I had no papers, FONASA, I had nothing.* (Venezuelan man Arica 1).

Several participants also reported having experienced xenophobic attitudes or having witnessed instances of xenophobia towards other migrants. *Specifically, I remember very well that I went to this gynecologist and of course he asked me my age, my name and then I suppose that in the file it says that I was Peruvian. And he made a pejorative comment like this: “ah, you’re peruanita”, just like that. And I said: “yes, I’m Peruvian.” And I remember very clearly that when I went to put on my robe so that he could examine me, he asked me: “and have you showered?”.* (Peruvian woman RM 1).

Some key actors also reported xenophobia: *There are doctors who are racist and it happens a lot to us, that we have to report that they did not want to treat them or, midwives that treat pregnant women badly*. (Key actor 1). Others highlighted the lack of skills and cross-cultural understanding *There was a Haitian mother who had twins, infants, one healthy and one sick, and that sick one in our ICU at the beginning of this year, and the team was very angry with the mother, because she did not come to see him. So, “she is not attached” […] and we already generated a meeting for that, and it is something super simple: can the mother enter with the other baby? No, she cannot enter the ICU with the other baby. Can the mother leave that nursing newborn to another caregiver? Difficult. If that mother leaves the infant at the neighbor’s, friend’s, child’s godmother’s house, what do we think: ohhh she is not attached to the infant, because she goes and leaves the baby with anyone* (Key actor 7).

Finally, systemic barriers stemming from the health system, emerged. They had to do with the availability of certain specialized care services in the public sector or with the perception that the private health system provides better quality care. *We have even talked about whether we could, because she undergoes some evaluation at the [public hospital] and a second evaluation is done at the [private clinic], so we are analyzing to see if it is possible that she improves at the [private clinic], to have greater peace of mind, after all, we are paying something to get better quality, better care* (Peruvian man RM 2).

In this context, due to not being able to access care in the public system or due to lack of availability, some participants seek care in the private system and mention the high cost of care as an important barrier. *In addition to this calculation, I had to undergo some gynecological exams and tried to find out how much these exams would cost me, and they would cost one hundred thousand, and it was extremely expensive, that is, for me it was unthinkable, of course, because I still didn’t have FONASA, they couldn’t give a discount, it was the price that it was. So, well, this day so far I haven’t taken the exams because I don’t have the money to pay for them and I haven’t found a way* (Colombian woman RM 2).

Notwithstanding the reported facilitators, mainly linked to enrollment in the health system, the general barriers identified are first linked to immigration status, information gaps, discrimination and xenophobia, lack of cross-cultural skills, lack of availability of certain care, negative perception of the public system, and costs in the private system. These are generally cross- sectional among the different study participants, migrants, civil society organizations, and health workers.

#### Specific Facilitators and Barriers to Access to Diagnosis and Treatment of COVID-19

Regarding accessing diagnosis and treatment of COVID-19, most of the participants were able to access PCR and treatment if required. However, some specific barriers emerged that led to not seeking a diagnosis or not accessing one. The first is related to current management recommendations at the time a diagnosis was required, where people with symptoms or close contact only had to isolate themselves. *We were already very ill, very high fever and everything; my sister-in-law called the doctor’s office and that’s when they told her no, that if we didn’t have symptoms of chest pain, if we didn’t feel short of breath, things like that, it wasn’t necessary to do any of that (PCR), we could treat it just with medication* (Cuban woman Antofagasta 1).

Similarly, one person mentioned the cost of voluntarily requesting a PCR as a barrier: *Some people went to have the PCR done but they did not want to do it because they were close contacts. […] The girls here who wanted to have the PCR done had to pay, to rule out that they had it, if I wanted to have it done I also had to pay twenty-five [thousand pesos], which I don’t have either, so of course, that was an issue.* (Colombian woman RM 2). On the other hand, some people did not seek a formal diagnosis when they were certain that they had been infected: *And my mom told me to go to the doctor, and if I could breathe I wouldn’t have to go, what were they going to tell me? if you have coronavirus and nothing else.* (Peruvian woman RM 2). In the same way, barriers related to cultural differences in the approach to the disease emerged, where, for example, a Haitian participant explained that some of his compatriots associate religion or being Afro-descendant with protection against contagion. *Because that is also a cultural thing on the inside, they also think, with COVID you can be praying to God and then you will be healthy, it is culture too, the same for not being infected, so it is something that they have on the inside, because of the color, because of race and there is no race at all, so these things I, a Haitian, have to fight with them* (Haitian man RM 1). The same participant refers to the stigmatization experienced by the Haitian community in relation to contagion: *A Haitian who had COVID, they accused him of having COVID and shoved him, because I saw that video, after that accusation they sent him to do the PCR, he did not have COVID so all the Haitians who saw that video are saying, no, that they are accusing us because we are brown, because we are Haitian* (Haitian man RM 1).

Similarly, key actors referred to significant communication barriers regarding diagnosis, especially those related to asymptomatic cases: *Now we have clearly seen ourselves facing important challenges, the difficulty in understanding the disease and the health management that made awareness, monitoring, and treatment more complex on many occasions. Think for a minute about the challenge it was to raise awareness of the asymptomatic for immigrant communities, that is, how to convey the message that someone, who is even feeling physically well, is still sick? So there is a very important culture shock, that is, let’s see, but I’m fine, why does he come to monitor me if I feel fine, I don’t have a fever, I don’t have a cough?* (Key actor 4).

Regarding treatment, some migrant participants mentioned the fear of not being treated in case they need it due to COVID-19, something that was also emphasized by the key actors as a central theme. *She did not go, she did not use the health system out of fear, she did not know if they were going to leave her there or, well, her fear was, being a foreigner, entering that health system and not being given the priority or the attention one deserves because of the fear of being a foreigner, me too, this is my only fear, of getting infected and entering a health system, a hospital, or something like that* (Venezuelan woman RM 2).

More specifically with regard to quarantine facilities, although they were well evaluated by a participant who used them, others mentioned difficulties related bring their children: *The other time they told me, the lady from FOSIS told me, if it were up to me they’re going to take you to a quarantine facility. But what am I going to do? Who am I going to leave the girls with?* (Peruvian woman Arica 1). The latter was emphasized by some key actors, who mentioned the impossibility for migrants in precarious housing situations to leave their belongings behind for fear of being robbed, as well as instances of discrimination or little cross-cultural adaptation initially. However, cultural and linguistic adaptations were valued to improve the strategy acceptability, such as when including Haitian doctors. *For example, the Haitians are working in some quarantine facilities to use them as a reference, it’s not that they’re just going to put Haitians there, but there will be Haitian professionals who speak the language* (Key actor 2).

During the pandemic, although no specific facilitators were identified, several of the participants mentioned having accessed diagnosis and treatment according to their needs. The barriers that emerged were cultural, related to experiences and/or anticipation of discrimination, socioeconomic issues, lack of support networks, and lack of information. The key actors interviewed placed special emphasis on communication and cultural barriers, including the approach to the disease, for access to diagnosis, treatment, or strategies such as quarantine facilities.

## DISCUSSION

The analysis presented in this article gives an account on the facilitators and barriers to access to health care for international migrants in Chile, both in a general and specific context during the COVID-19 pandemic. It focuses on the experiences and perceptions of 30 migrants and 10 key actors in the social and health area, interviewed in November and December 2020, almost a year after the pandemic began. At that time, Chile was facing significant challenges in managing the pandemic, with consequences for sectors of the population under social vulnerability conditions, among which are international migrants. In the context of a pandemic, questions arise regarding effective access to health care in this population group that has been facing challenges in guaranteeing their right to health, and it becomes relevant to investigate the access facilitators and barriers experienced by international migrants and observed by various key actors, to contribute to improving access and care.

The facilitators identified in the general context are related to the role of formal employment in triggering enrollment in the health system, either public or private, which, in its turn, contributes to facilitating access to care. On the other hand, having, in their support network, people already registered in the system and with experience as users facilitates registration and subsequent use, as well as having had good experience with health care facilitates continuity of use. The first facilitator is closely linked to the migratory situation, since only people with a regular migratory situation can access formal employment. The regularization of the migratory and labor status of migrants is then a central element to facilitate registration in the health system^(16)^. This, likewise, is reinforced with the report of the migratory situation as the first barrier to access to health services, something widely documented in the existing literature at a global level^(17,18,19)^. In Chile, however, there is a legal framework that allows care regardless of immigration status, which suggests the urgent need to reinforce information about the rights of migrants, both among health system professionals and among the country’s migrant communities^(20,21)^. Moreover, the information obtained from relatives and/or acquaintances who are already affiliated emphasizes the importance of support networks for access to health coverage in the settlement phase, and the availability of linguistically and culturally relevant information to promote the registration to the health system^(22,23)^.

Another important barrier identified in this study is also linked to the dissemination of information during the pandemic, where erroneous information circulated about contagion and the Haitian community was accused of circulating the virus, discouraging the search for diagnosis and treatment. In this regard, the timely dissemination of official information regarding the right to health of migrants in the context of a pandemic and the mechanisms of contagion can help avoid situations of discrimination, stigmatization, dispel the fears of migrants of not being attended if necessary, and promote adherence to measures to prevent infection and treatment. This is also part of a broader context of xenophobia and discrimination observed in health care in general, both in the present study and in previous studies carried out in Chile^(24,25)^, but also in other areas of daily life in the country^(26)^.

On the other hand, a lack of comprehension of the cross- cultural and socioeconomic context of migrants was reported, both in the general context of health care, and in certain pandemic management strategies such as quarantine facilities, which hinder their acceptability and effective use, suggesting the need for greater awareness and training of health system actors, from authorities to teams at all levels of care. This phenomenon has also been observed in other studies in Chile and globally^(24,27,28)^.

Finally, barriers related to the lack of availability of certain benefits in the public system and the costs associated with seeking care in the private system were identified, which is related to broader inequities in the Chilean health system, which are also faced by local people^(29)^. This is due to the health system fragmentation, where there coexists, on the one hand, a public system facing overload and difficulties in responding to the demands and needs of its users who generally belong to the most vulnerable socioeconomic strata and with a high prevalence of chronic diseases. On the other hand, a private system that prioritizes the incorporation of patients of high socioeconomic level without pre-existing disease with high costs associated with both anticipation and care^(13,29)^. Eliminating these inequities requires an approach that aims at a deep and structural change to promote health of all the people present in the national territory.

The barriers and facilitators for access to health for international migrants identified in this study are connected to the social determinants of health and the social vulnerability faced by migrants in Chile, hindering the fulfilment of their right to health, guaranteed both by international human rights frameworks and by the national legal framework^(30)^. This, in the context of the COVID-19 pandemic and the promotion of public health, becomes an urgent challenge to be solved in an intersectoral manner. This exploratory study is therefore relevant for the health sector and for decision makers and policy makers at the national level. The main limitation of the study is that it included only Spanish-speaking participants with Internet access, leaving participants who potentially face high social vulnerability out of the sample.

## CONCLUSIONS

International migrants face situations of social and rights violations that impact their health, particularly with regard to access to health care. The facilitators and barriers revealed from the voice of relevant actors in this study are connected with migratory status, support networks, access to linguistically and culturally relevant information, positive or negative experiences (discrimination, xenophobia) in health care, the cross-cultural relevance of care and structural inequities typical of the Chilean health system. Although all migrants do not face the same degree of social vulnerability, taking into account the diversity of migratory trajectories and socioeconomic contexts of the migrant population in Chile, migration is recognized as a social determinant of health and strategies that allow breaking different barriers of access to the health system in this population shall be developed, based on an cross-cultural, Human Rights, and social justice approach. Thus, considering that the barriers identified are also observed in other countries, some being intermediate transit countries for migrants arriving in Chile, regional and global cooperation is required to improve access to health services regardless of the migratory status.
