# ERRATUM: Access to health services for international migrants during the COVID-19 pandemic: a qualitative study

**DOI:** 10.1590/1980-220X-REEUSP-2022-0443eren

**Published:** 2024-02-26

In the article “Access to health services for international migrants during the COVID-19 pandemic: a qualitative study”, with DOI code number: https://doi.org/10.1590/1980-220X-REEUSP-2022-0443en, published at Revista da Escola de Enfermagem da USP [online], v. 57 (spe): e20220443, on the page 7:

**Where it was written:**

**DESCRIPTORS**

Human Migration; Health Services; COVID-19; Chile.

**Should read:**

**DESCRIPTORS**

Human Migration; Health Services Accessibility; COVID-19; Chile.

**Where it was written:**

**DESCRITORES**

Migração Humana; Serviços de Saúde.

**Should read:**

**DESCRITORES**

Migração Humana; Acesso aos Serviços de Saúde; COVID-19; Chile.

**Where it was written:**

**DESCRIPTORES**

Migración Humana; Servicios de Salud; COVID-19; Chile.

**Should read:**

**DESCRIPTORES**

Migración Humana; Accesibilidad a los Servicios de Salud; COVID-19; Chile.

